# A middle-aged male patient with de Winter syndrome: a case report

**DOI:** 10.1186/s12872-020-01619-6

**Published:** 2020-07-18

**Authors:** Bo Lu, Deyu Fu, Xunjie Zhou, Mingtai Gui, Lei Yao, Jianhua Li

**Affiliations:** Department of Cardiology, Yueyang Hospital of Integrated Traditional Chinese and Western Medicine, Shanghai University of Traditional Chinese Medicine, No. 110 Ganhe Road, Hongkou District, Shanghai, 200437 China

**Keywords:** Electrocardiogram, ST-elevated myocardial infarction, Myocardial infarction, Revascularization

## Abstract

**Background:**

De Winter syndrome accounts for approximately 2% of all patients with acute anterior myocardial infarction admitted to the emergency department, and is characterized by severe stenosis of the left anterior descending coronary artery (LAD). The ECG changes are not recognized by ECG software, and poor understanding of the syndrome among physicians may lead to misdiagnosis, delayed reperfusion, and mortality.

**Case presentation:**

A 51-year-old male patient presented with a newly developed ECG pattern suggestive of de Winter Syndrome. Coronary angiography revealed anterior myocardial infarction. Based on the ECG and clinical manifestations, the patient was diagnosed with de Winter syndrome and underwent timely percutaneous coronary intervention to revascularize the left anterior descending artery (LAD). The patient showed good outcomes and no complications at 4 months after the operation.

**Conclusions:**

This case highlights the importance of being aware of the possibility of de Winter syndrome in patients with symptoms of myocardial infarction but atypical ECG in order to conduct early revascularization and treatments.

## Background

Electrocardiogram (ECG) remains crucial in the diagnosis of myocardial infarction because of its convenience, quickness, and accuracy. Timely identification of ECG signs of acute coronary occlusion is necessary for timely restoration of blood flow and improvement of patient outcomes [1]. ST-segment elevation or new left bundle branch block is generally considered to be the most common ECG change in the presence of acute coronary occlusion. Nevertheless, misdiagnosis or missed diagnosis is clinically reported in about 30% of the patients with acute coronary occlusion due to atypical ECG, leading to serious consequences.

De Winter syndrome is among the presentations that can be misdiagnosed on ECG. De Winter et al. [2] reviewed the ECG of 1532 patients with acute coronary syndrome with signs of occlusion of the proximal left anterior descending artery (LAD) and found that 30 of them (2.0%) did not display the super-acute myocardial infarction ECG changes with typical ST-segment elevation. Their ECG showed that the J point of leads V1-V6 was lowered by 1–3 mm, and the ST segment appeared as an upsloping depression, followed by tall, positively symmetrical T waves. The QRS complexes were usually not widened or were only slightly widened. In some patients, there was a loss of precordial R-wave progression. The aVR lead showed slight ST-segment elevation in most cases [3]. This ECG pattern was later named de Winter syndrome. Despite being atypical in patients with typical ST-elevated myocardial infarction (STEMI), this ECG pattern has a 100% positive predictive rate for myocardial infarction, and the culprit vessel is the proximal LAD, which can cause extensive anterior myocardial infarction. De Winter syndrome is a rare ECG pattern with important values of localization and qualitative diagnosis. Nevertheless, ECG diagnostic software usually cannot identify this pattern. Therefore, the early diagnosis of these patients by the emergency physician is essential and directly affects whether the patient should undergo emergency reperfusion therapy. In the present study, a patient with de Winter syndrome is reported.

## Case presentation

The patient provided informed consent for the presentation of his case. The Ethics Committee of Yueyang Hospital approved this case report. A 51-year-old male patient was admitted to the Emergency Department of Yueyang Hospital of Integrated Traditional Chinese and Western Medicine, Shanghai University of Traditional Chinese Medicine, because of soreness of both shoulders for 2 days accompanied by chest tightness and pain for 5 h. Two days before admission, slight soreness of both shoulders was reported due to excessive fatigue, but without chest tightness, chest pain, dizziness, and palpitation. He sought no treatment at that time. At about 4.5 h before admission, severe chest tightness and pain began abruptly, but without cold sweats, near-death experience, nausea, and vomiting.

The patient was previously healthy and had no known history of chronic diseases such as hypertension, diabetes, and heart diseases. He was a current smoker with a 30-year-history of smoking 30 cigarettes per day. Physical examination showed body temperature of 36.6 °C, pulse at 78 beats/min, respiratory rate at 18 breaths/min, and blood pressure at 110/72 mmHg (1 mmHg = 0.133 kPa). He had a clear consciousness and regular heart rhythm with normal heart sounds. No cardiac murmurs were heard on auscultation. The results of the lung and abdomen examinations were unremarkable.

**Fig. 1:**
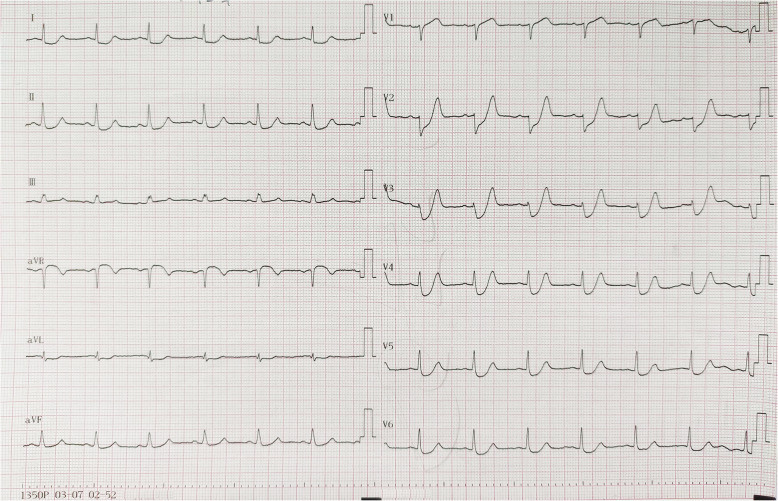
Emergency ECG showing de Winter ECG pattern

At 10 min into admission, emergency ECG (Fig. 1) revealed sinus rhythm, upsloping ST-segment depression of 0.1–0.4 mV in leads V2-V6, horizontal ST-segment depression in leads I, II, aVL, III, and aVF, tall-peaked T wave in leads V2-V4, and ST-segment elevation of about 0.15–0.2 mV in the aVR lead. The ECG machine read the ECG as being normal, but those changes were characteristics of de Winter syndrome. Point-of-care testing (POCT) showed: D-dimer at < 0.1 mg/L (reference value < 0.5 mg/L), creatinine kinase (CK)-MB at 6.4 ng/ml (reference value < 5 ng/ml), troponin I at 0.16 ng/ml (reference value < 1 ng/ml), myoglobin at 165.6 ng/ml (reference value < 20 ng/ml), and N-terminal-pro hormone brain natriuretic peptide (NT-proBNP) at < 300 pg/ml (reference value < 300 pg/mL). Treatment with ticagrelor 180 mg (chewing) and 300 mg aspirin tablets (chewing) was performed immediately.

**Fig. 2:**
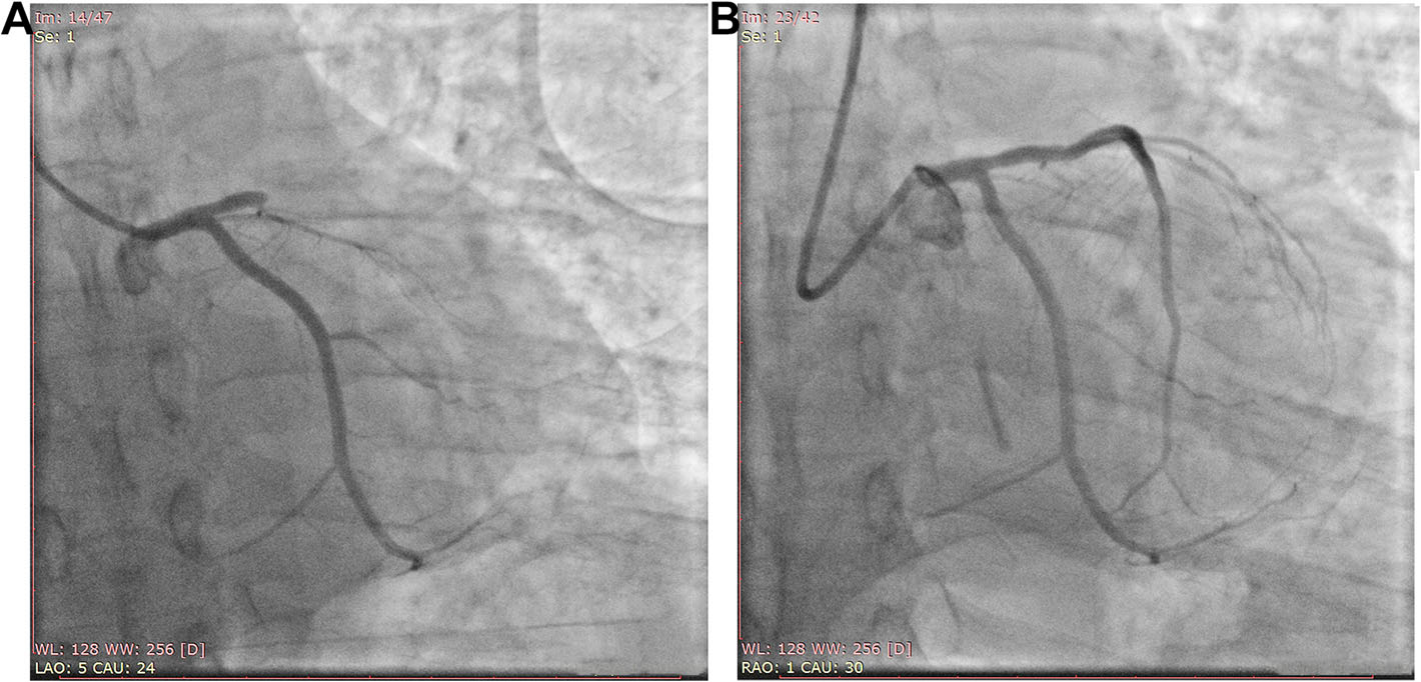
Angiography results before (**a**) and 8 days after (**b**) stent implantation

The patient still complained of persistent chest pain, and emergency coronary angiography was immediately performed through the fast track of the Chest Pain Center. The results revealed no stenosis of the left main coronary artery, but complete occlusion of the proximal LAD, with TIMI flow grade 0 (Fig. 2a and Supplementary materials, Video 1). No significant stenosis was seen in the left circumflex coronary artery. The right coronary artery was slightly irregular but without significant stenosis. By combining the results of ECG and angiography, the LAD was considered the culprit vessel. After aspiration of the thrombus, a stent was implanted (Supplementary materials, Video 2). Eight days after implantation, the stent was post-expanded to reduce the risk of no-reflow and to ensure the best release of the stent (Fig. 2b, Supplementary materials, Videos 3, 4 and 5).

**Additional file 1.** Angiography was performed in the patient with de Winter syndrome; Video 1, revealing no stenosis of the left main coronary artery, but complete occlusion of the proximal LAD, with TIMI flow grade 0.

**Fig. 3:**
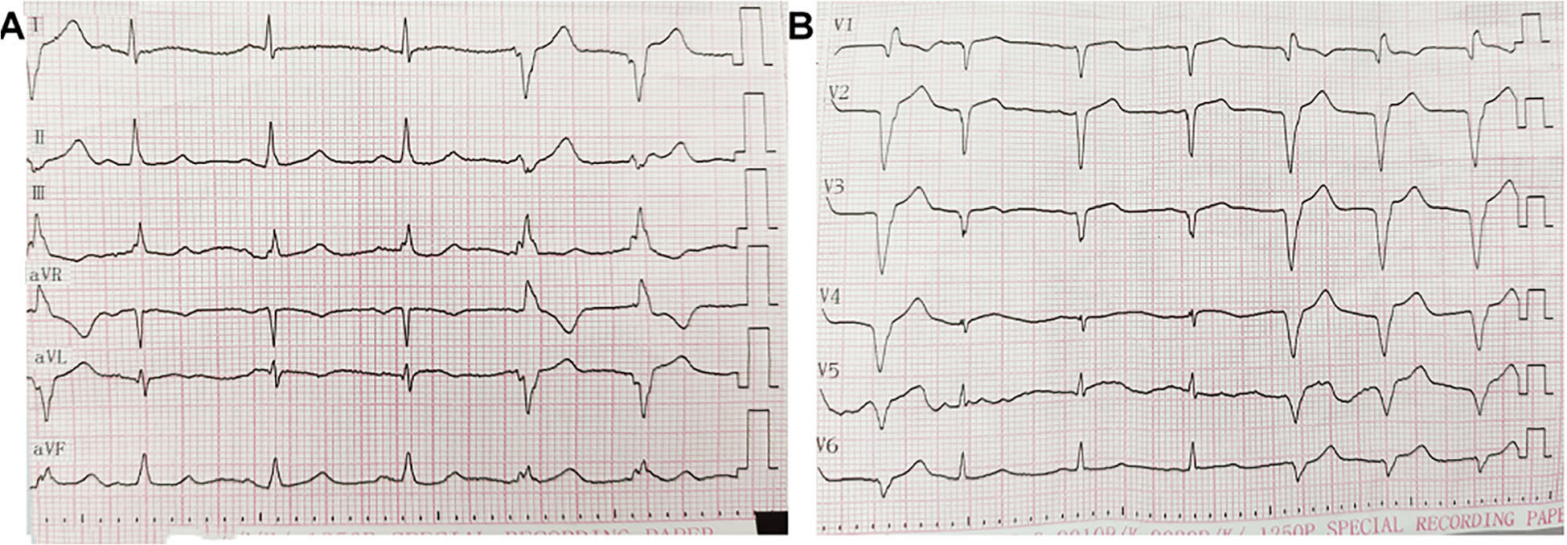
ECG immediately after stent implantation

Immediately after the operation, the ECG characteristics of de Winter Syndrome disappeared, and there was accelerated idioventricular rhythm caused by reperfusion. Pathological Q waves were observed in the precordial leads V1-V3, and the T-waves in the precordial leads showed dynamic changes. The T-waves in leads V4-V6 were low and flat (Fig. 3).

Transthoracic echocardiography performed at 11.5 h into admission revealed multiple segmental ventricular wall motion abnormalities in the left ventricle (anterior wall, lateral wall, ventricular septum, and apex), small regurgitation of the mitral and tricuspid valves, decreased left ventricular systolic function (ejection fraction is 48%) and decreased left ventricular compliance (Supplementary materials, Videos 6, 7 and 8). The patient developed decreased cardiac systolic function.

Aspirin enteric-coated tablets (100 mg/d, po) and ticagrelor (180 mg/d, po) were given for anti-platelet therapy. Atorvastatin (20 mg/n, po) was given for lipid lowering and plaque consolidation. Metoprolol (23.75 mg/d, po) was given to control the heart rate.

**Fig. 4:**
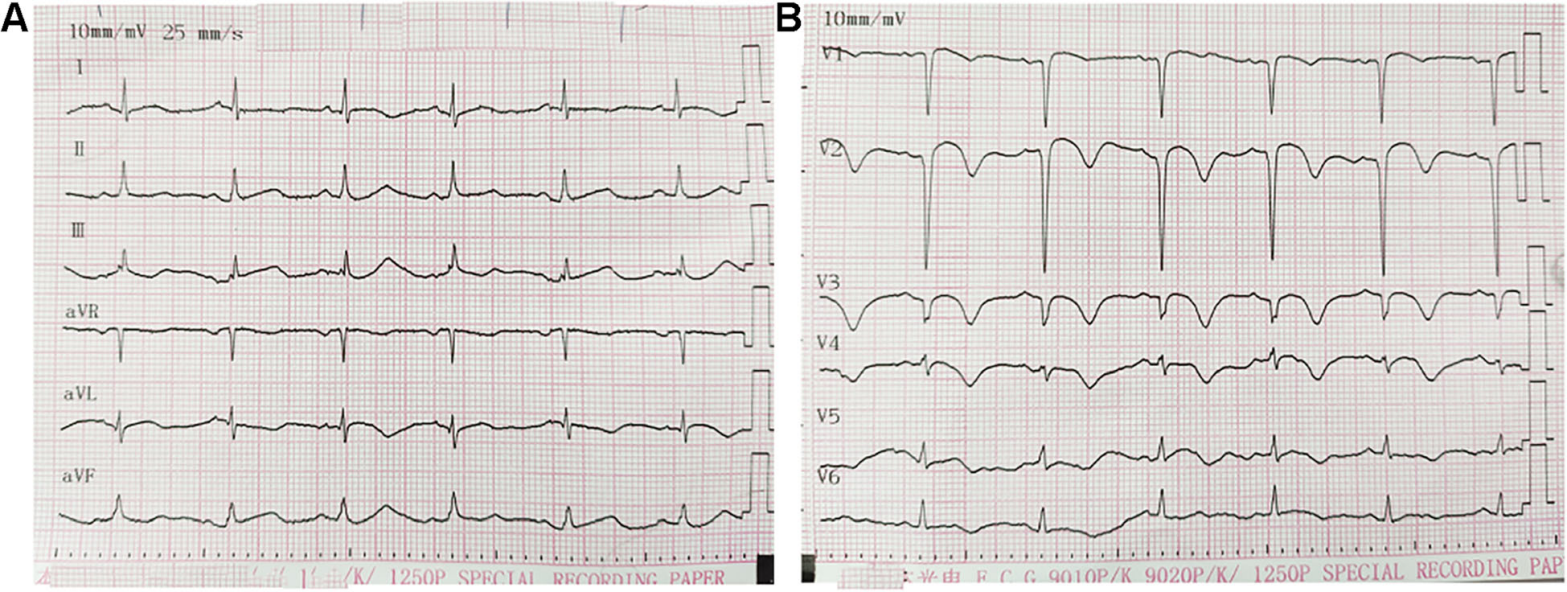
ECG 3 days after stent implantation

The ECG 3 days after implantation showed that the T-waves were inverted in the precordial leads (Fig. 4). The ECG was now typical of anterior STEMI. The patient was discharged 9 days after implantation. There was no obvious discomfort during follow-up.

## Discussion and conclusions

Acute myocardial infarction has a high mortality rate, but not all patients with acute coronary occlusion show ST-segment elevation on the ECG. This type of ECG is called “STEMI-equivalent” and also requires emergency percutaneous coronary intervention. At present, the definition of STEMI-equivalent is not mentioned in the latest guidelines from the European Society of Cardiology and the American Heart Association, but the Fourth Universal Definition of Myocardial Infarction proposed that attention should be paid to ECG signs that can increase the likelihood of a diagnosis of myocardial infarction, such as de Winter syndrome. Patients with acute chest pain accompanied by a de Winter-like ECG is considered a STEMI-equivalent, suggesting occlusion or severe stenosis of the proximal LAD [4, 5]. In the case of de Winter syndrome, the majority are solitary LAD lesions, and there is no obvious coronary artery lesion in other locations [3]. Verouden et al. observed that although no patient characteristics could be associated with de Winter syndrome, most patients were young males with hypercholesterolemia [3]. In addition, they observed a “wrap-around” LAD artery in about 50% of the patients that are usually associated with ST-segment elevation in precordial and inferior leads, but that is not associated with an absence of the ST-elevation [3]. Hence, Verouden et al. [3] hypothesized that this could be due to the large area of transmural ischemia, without injury currents being generated and picked up by the precordial leads, but only currents directed upwards to the aVR lead. They also hypothesized that the lack of ST-elevation could be due to the lack of activation of the ATP-sensitive potassium channels [3]. Nevertheless, the exact mechanisms leading to de Winter ECG remain to be determined.

Although de Winter ECG does not show ST-segment elevation, it indicates acute coronary occlusion. Recent studies suggested that de Winter syndrome may be a transient change in the progression of the acute coronary syndrome, and it could eventually change into a typical STEMI ECG [4, 6] (as in the case reported here) or even into a normal ECG [7]. Therefore, if the ECG is not monitored in time, the ECG changes of de Winter syndrome cannot be observed. In the case presented here, even if the ECG changed into a typical STEMI ECG, a clinical progression to STEMI was not observed in the patient.

The misdiagnosis rate is extremely high because of the atypical ECG. Therefore, the therapeutic window might be missed [8]. Since this type of ECG has no ST-segment elevation, clinicians often have a poor understanding of it, ignoring its risks and categorizing it as NSTEMI, which further delays the diagnosis and treatment [9, 10]. Furthermore, in the case reported here, the biomarkers could not be relied upon for the diagnosis of myocardial infarction. Indeed, troponin I and NT-proBNP were not elevated, and CK-MB was slightly elevated; only myoglobin levels were markedly elevated. This poses an additional challenge for the cardiologist since the biomarker panel does not readily suggest myocardial infarction, which, in the presence of a non-typical ECG, could lead to missed or delayed diagnosis. Here, the symptoms and ECG prompted the physician to perform angiography, which showed LAD occlusion. Coronary angiography should be performed in time to identify the occluded blood vessels to protect the myocardium from ischemic necrosis.

The Chinese Guidelines for Percutaneous Coronary Intervention (2016) proposed that shortening the time delay in STEMI patients was the key to performing reperfusion therapy and that the door-to-balloon time should be < 90 min [11]. The diagnosis and treatment of patients with de Winter syndrome are similar to the treatment of STEMI since there is an actual occlusion that needs to be managed in a timely manner.

This case highlights the importance of being aware of the possibility of de Winter syndrome in patients with symptoms of myocardial infarction but atypical ECG in order to conduct early revascularization and treatments.

## Supplementary information

**Additional file 2.** No significant stenosis was seen in the left circumflex coronary artery. The LAD was considered the culprit vessel. After aspiration of the thrombus, a stent was implanted.

**Additional file 3.** Eight days after implantation, the stent was post-expanded.

**Additional file 4.** Eight days after implantation, the stent was post-expanded.

**Additional file 5.** Eight days after implantation, the stent was post-expanded.

**Additional file 6.** Echocardiography of the patient with de Winter syndrome. Transthoracic echocardiography performed at 11.5 h into admission revealed multiple segmental ventricular wall motion abnormalities in the left ventricle (anterior wall, lateral wall, ventricular septum, and apex).

**Additional file 7.** Echocardiography of the patient with de Winter syndrome. Transthoracic echocardiography performed at 11.5 h into admission revealed multiple segmental ventricular wall motion abnormalities in the left ventricle (anterior wall, lateral wall, ventricular septum, and apex).

**Additional file 8.** Echocardiography of the patient with de Winter syndrome. Transthoracic echocardiography performed at 11.5 h into admission revealed multiple segmental ventricular wall motion abnormalities in the left ventricle (anterior wall, lateral wall, ventricular septum, and apex).

## Data Availability

The datasets used and/or analyzed during the current study are available from the corresponding author on reasonable request.

## Abbreviations

ECG: Electrocardiogram
LAD: Left anterior descending coronary artery
STEMI: ST-elevated myocardial infarction
